# Tracing Management and Epidemiological Characteristics of COVID-19 Close Contacts in Cities Around Chengdu, China

**DOI:** 10.3389/fpubh.2021.645798

**Published:** 2021-12-15

**Authors:** Kai Yang, Jiali Deng, Liang Wang, Shan Jiang, Rong Lu, Zhijian Liu, Xiaoli Tuo

**Affiliations:** ^1^Emergency and Business Management Office, Chengdu Center for Disease Control and Prevention, Chengdu, China; ^2^Department of Orthopaedics, Chengdu Medical College, Chengdu, China; ^3^Department of Infectious Disease Control, Center for Disease Control and Prevention of Chenghua District, Chengdu, China

**Keywords:** close contact, COVID-19, epidemiological, epidemic prevention and control, tracking

## Abstract

**Introduction:** Close contacts have become a potential threat to the spread of coronavirus disease 2019 (COVID-19). The purpose of this study was to understand the epidemiological characteristics of close contacts of confirmed or suspected cases of COVID-19 in the surrounding cities of Chengdu, China, so as to provide a basis for the management strategy of close contacts.

**Methods:** Close contacts were determined through epidemiological investigation of indicated cases, and relevant information was entered in the “Close Contact Information Management System.” Retrospective data of close contacts from January 22 to May 1, 2020 were collected and organized. Meanwhile, the contact mode, isolation mode, and medical outcome of close contacts were descriptively analyzed.

**Results:** A total of 986 close contacts were effectively traced, with an average age of (36.69 ± 16.86) years old, who were mainly distributed in cities of eastern Chengdu. The frequency of contact was mainly occasional contact, 80.42% of them were relatives and public transportation personnel. Besides, the time of tracking close contacts and feedback was (10.64 ± 5.52) and (7.19 ± 6.11) days, respectively. A total of seven close contacts were converted to confirmed cases.

**Conclusions:** Close contacts of COVID-19 have a risk of invisible infection. Early control of close contacts may be helpful to control the epidemic of COVID-19.

## Introduction

In December 2019, a series of unexplained pneumonia cases appeared in Wuhan, Hubei, China (1–3), which was subsequently identified by etiological identification as a novel coronavirus, named the coronavirus disease 2019 (COVID-19). Although many details of the emergence of the virus are still unknown, several pieces of evidence have confirmed human-to-human transmission (4–6). Afterwards, the World Health Organization (WHO) announced it as a Public Health Emergency of International Concern (PHEIC) on January 30, 2020 (7). COVID-19 has spread worldwide, which has caused more than 239 million cases and 4.87 million deaths as of October 18, 2021.

To control the further spread of the epidemic, the Wuhan government has implemented a “lockdown” (8). Unfortunately, this period coincides with the traditional mass movement before the Spring Festival, that is, a form of “going home.” As a result, more than 5 million people have left Wuhan, which undoubtedly increased the risk of infection in other areas (9). Chengdu, located in the southwest of China, is an important transportation hub in China. The increase in population mobility also increased the import of infectious diseases. Since the first COVID-19 case reported in Chengdu on January 22, there have been 166 cases as of May 1, 2020. Existing data showed that the epidemic of COVID-19 in Chengdu was dominated by imported cases, and most patients were close contacts of confirmed cases, that is, “second-generation cases.” How to “contain” the “three links” of infectious diseases and timely and accurate detection and tracking of close contacts are still a major focus and difficulty in epidemic control.

Close contact tracing is an intervention that requires the index case to provide as much information as possible about contacts who have acquired the risk of infection within a given period of time before the test results are available (10). Close contact management has become one of the core strategies to reduce additional transmission (11). Jing et al. (12) have provided important insights into the factors affecting the transmission of COVID-19 primary cases and the susceptibility of their close contacts.

Existing studies have confirmed that effective concentration or home isolation of close contacts could restrain the spread of COVID-19 to a certain extent, which could also create a good living and development environment (11, 13). At the same time, collecting accurate epidemiological data through contact tracing can increase the awareness of the epidemic and draw up effective intervention measures.

Since the outbreak of the COVID-19 epidemic, Chengdu has adopted strict case isolation treatment, close contact tracing, and medical observation measures, which have effectively prevented the spread of the epidemic. From the perspective of close contact tracing, this study aims to understand the epidemiological characteristics and tracing management of close contacts transferred from Chengdu to surrounding cities, and at the same time, scientifically and reasonably determine the quarantine objects, so as to provide a basis for epidemic prevention and control.

## Materials and Methods

### Data Collection

Close contacts were determined following the “Management Plan of Close Contacts of COVID-19 Cases” in the “COVID-19 Prevention and Control Program” of the China Health Commission (14). Possible close contacts were determined through epidemiological investigation of confirmed, asymptomatic, or suspected cases. Moreover, some of the information on close contacts came from the personnel of public security, tourism, and other departments or areas who request assistance in the investigation.

Close contacts refer to people who have not had effective protection from suspected or confirmed cases (within 1 meter) from 2 days before symptoms appear, or 2 days before sampling asymptomatic samples, including people who are living together, studying together, those under diagnosis and treatment, and those sharing transportation, etc. Relevant information was entered into the “Close Contact Information Management System” by health workers. Retrospective data of close contacts from January 22 to May 1, 2020 were used in our analysis.

### Close Contact Management Measures

According to the distribution of close contacts in the inner districts and counties of Chengdu, the basic information of close contacts was entered into the system by the prevention and control personnel of the local jurisdiction. Once after identification, the close contacts were subjected to centralized or home isolation for medical observation for 14 days; body temperature and respiratory symptom were monitored approximately every day. In addition, the SARS-CoV-2 nucleic acid test was performed at least twice before the quarantine was ended, with an interval of more than 24 h each time. If there was no abnormality, isolation was terminated.

### Research Content

We collected the information of close contacts among people who were isolated due to COVID-19, which included the basic information of close contacts, relationship with original cases, mode of isolation observation, mode of contact, location of contact, and presence of clinical symptoms, etc.

### Statistical Analysis

The retrospective data and relevant information of the close contacts were collected through the “Close Contact Information Management System” and the database was established. Data were statistically sorted and analyzed by SPSS version 22.0 software (IBM Corp, NY, USA). The qualitative data were statistically described by frequency, composition ratio or rate, and statistically analyzed by chi-square test. P < 0.05 was considered statistically significant. ArcGIS version 10.5 software (Environmental Systems Research Institute, Redlands, CA, USA) was used to describe the spatial distribution of close contacts.

## Results

### Screening of Close Contacts

**Figure 1:**
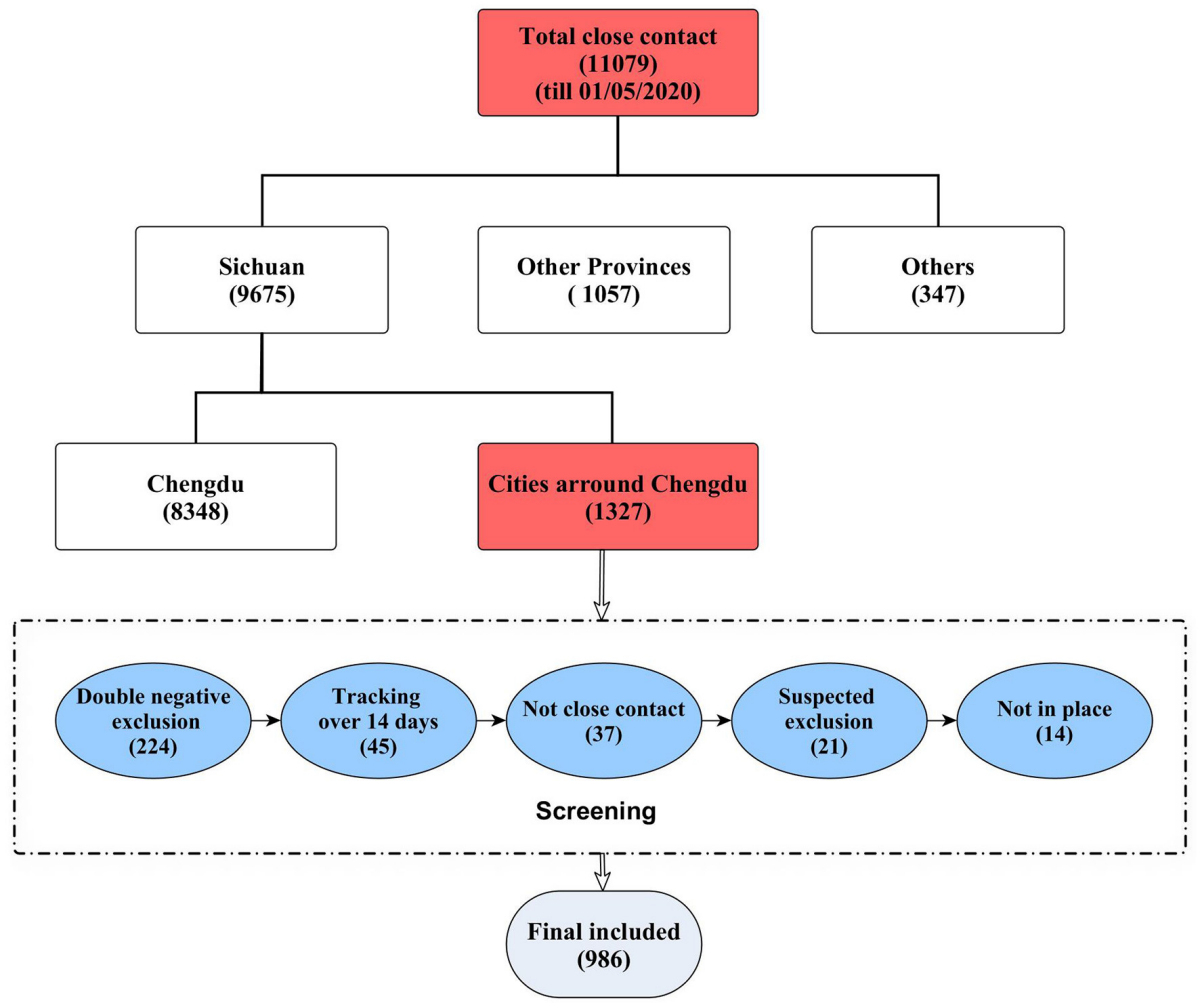
The flow chart of close contact screening.

According to the epidemiological investigation, 11,079 close contacts were tracked by May 1, 2020. Among them, 8,348 cases were local management close contacts in Chengdu and 1,057 cases were in other provinces. Through further screening of close contacts in cities around Chengdu, 986 cases were finally included in this study (details are shown in Figure 1).

### Distribution Characteristics of Close Contacts

**Figure 2:**
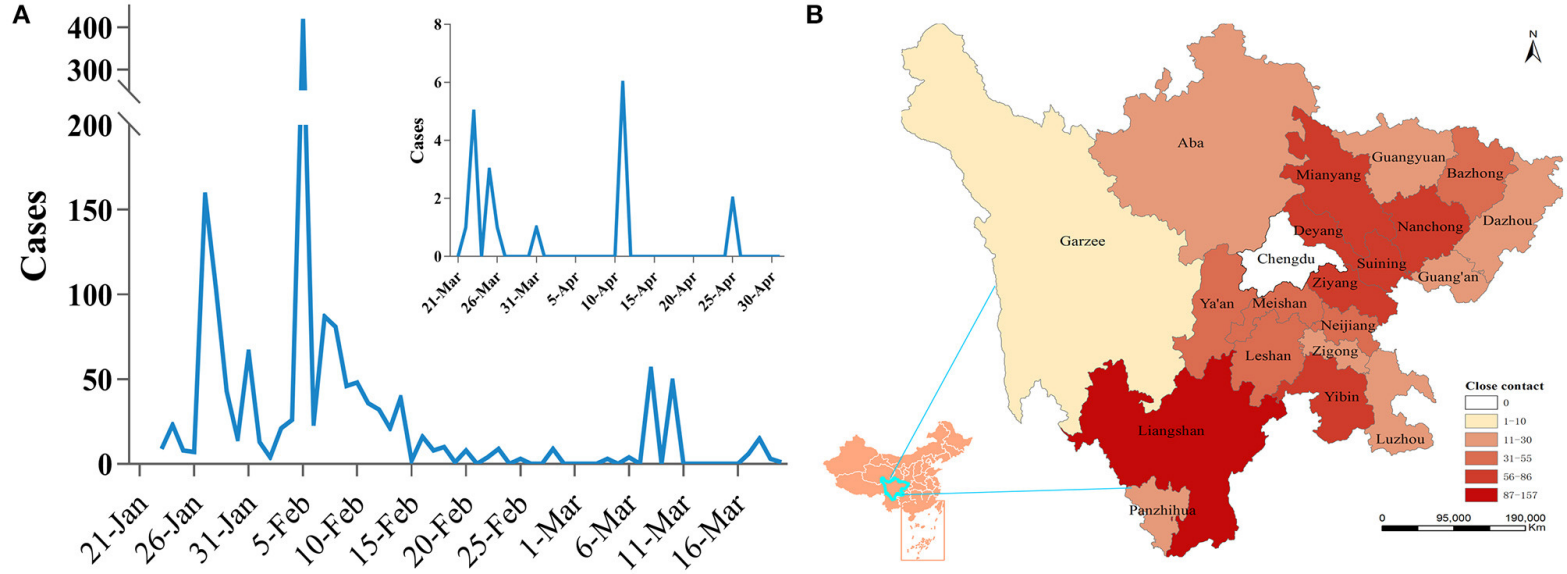
The distribution characteristics of close contacts. **(A)** Time distribution of close contacts. **(B)** Spatial distribution of close contacts.

Through the analysis of the discovery time of all close contacts, the distribution presented as three different peaks, which were mainly concentrated from January 26 to February 14 (accounting for 82.85%). After a stable period of nearly 20 days, another surge appeared on March 8 and then stabilized again (Figure 2A). A total of 986 close contacts were distributed in 20 cities around Chengdu (ranging from 1 to 157, average: 46.96). Except for the Liangshan Prefecture, the cities with more close contacts were mainly located in the eastern part of Chengdu, accounting for 51.52% (Figure 2B).

### Basic Characteristics of Close Contacts

Among the close contacts, there were 558 men and 428 women, with a male:female ratio of 1.30:1. The average age of close contacts was (36.69 ± 16.86) years old, which mainly concentrated in the age group of 15–60 years (79.72%); no significant difference was found in the distribution of different ages (*P* < 0.001). The frequency of contact between close contacts and cases was mainly occasional contact (60.75%), and the relationship with cases was mainly relatives (30.12%) and co-passengers (50.30%), and most of them were in the same train compartment (70.35%, data not shown). Contact places were mainly residential and transportation (81.54%), and the method of contact was mainly sharing rides and gatherings (71.70%), while hospital contact accounted for about 9.53% (Table 1).

**Table 1:** The basic characteristics of close contacts.

**Index**	**Cases (*n*%)**	**χ^2^**	***P*-value**
**Gender**			
Male	558 (56.59)	17.53	<0.001
Female	428 (43.41)		
**Age**			
0– <15	87 (8.82)		
15– <30	256 (25.96)		
30– <45	226 (22.92)	2399.19	<0.001
45– <60	304 (30.83)		
≥60	113 (11.46)		
**Relationship with cases**			
Relatives	297 (30.12)	692.84	<0.001
Fellow passengers	496 (50.30)		
Colleague	23 (2.33)		
Diagnosis	32 (3.25)		
Others	138 (14.00)		
**Personnel classification**			
Medical staff	20 (2.03)	1836.58	<0.001
Non-medical staff	966 (97.97)		
**Contact frequency**			
Occasionally	599 (60.75)	789.33	<0.001
General	207 (20.99)		
Often	180 (18.26)		
**Contact location**			
Domicile	295 (29.92)	773.85	<0.001
Restaurant	23 (2.33)		
Vehicle	496 (50.30)		
Hospital	94 (9.53)		
Others	78 (7.91)		
**Contact mode**			
Vehicle	496 (50.30)	627.5	<0.001
Dinner together	211 (21.40)		
Domesticity	136 (13.79)		
Diagnosis and treatment	132 (13.39)		
Others	11 (1.12)		
**Isolation mode**			
Centralized isolation	712 (72.21)	1865.32	<0.001
Home isolation	236 (23.94)		
Hospital treatment	6 (0.61)		
Other	32 (3.25)		

### Time Index Analysis

Through the analysis of time indexes of close contacts, it was found that the time of tracking close contacts was (10.68 ± 5.46) days, and the feedback time of other cities after receiving assistance in the investigation was (7.24 ± 6.14) days. The time from case discovery to close contacts release was (4.81 ± 4.14) days, which was longer than the actual isolation time (4.17 ± 4.40; *t* = 3.175, *P* = 0.002; Table 2).

**Table 2:** Close contact discovery and isolation time.

**Index**	**Time (days)**	**P_25_**	**P_75_**
Close contact tracking time	10.64 ± 5.52	6.31	15.00
Feedback time	7.19 ± 6.11	7.00	11.00
Supposed isolation time	4.81 ± 4.14	1.00	7.69
Actual isolation time	4.17 ± 4.40	3.00	5.00

*Data were expressed as mean ± S.D, and the 25th and 75th percentiles were described*.

### Outcome of Close Contacts

**Figure 3:**
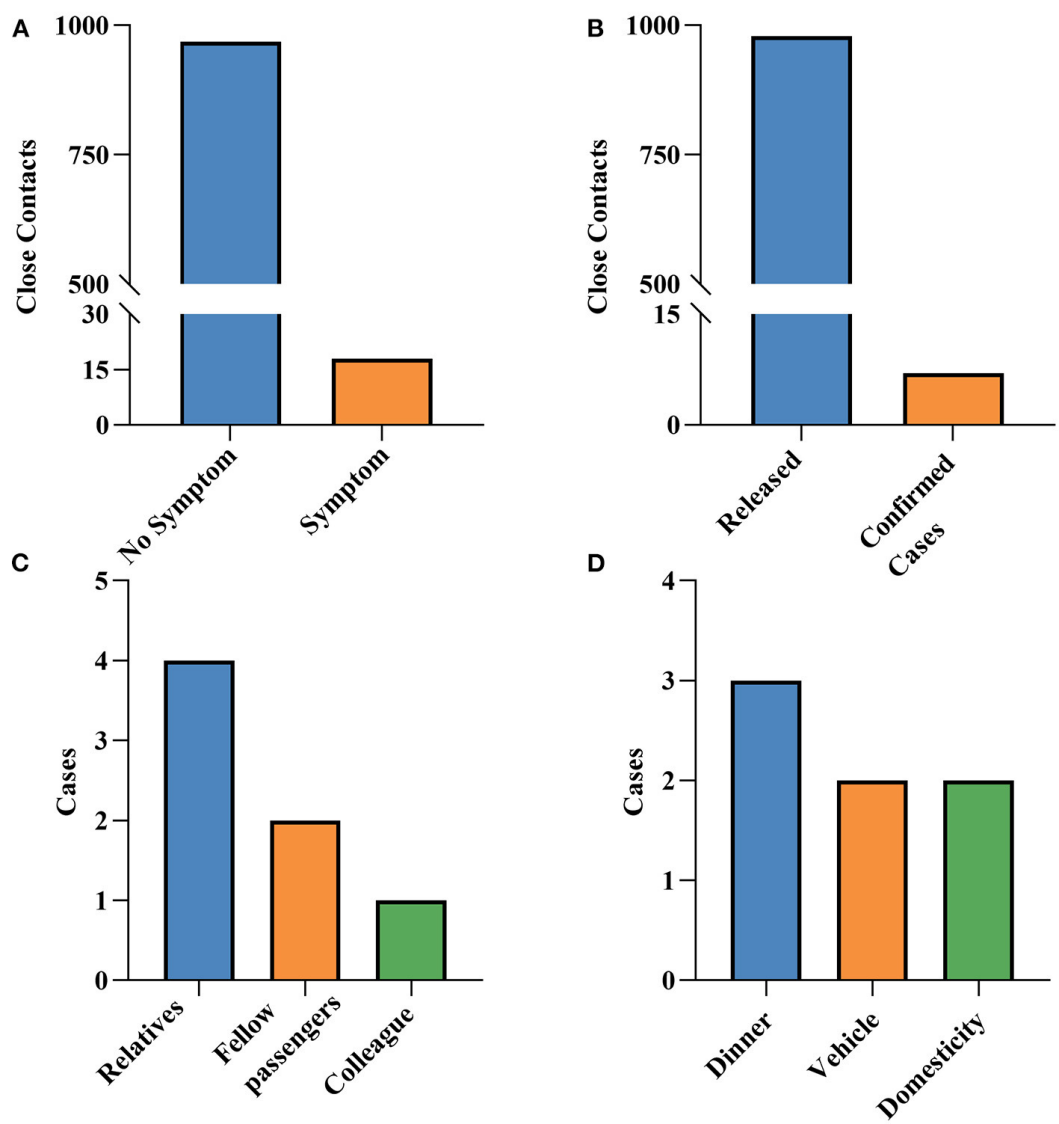
The outcome of close contacts. **(A)** Whether they have symptoms or not. **(B)** Status of close contacts. **(C)** Relationship with indexed cases. **(D)** Contact mode with indexed cases.

Among the 986 close contacts, 18 had symptoms, mainly manifested as upper respiratory symptoms such as cough, runny nose, and sore throat (data not shown). A total of seven close contacts were converted to confirmed cases, with the majority of them frequent contacts (42.86%). Meanwhile, the seven cases were mainly the relatives and co-passengers of indicated cases, and the main contact mode was eating together (42.86%; Figure 3).

## Discussion

COVID-19 has caused a widespread pandemic, and human-to-human transmission was discovered as early as the beginning of the epidemic (5, 15, 16). With the increase in population mobility, it is undoubtedly possible for the disease to spread further. As for the close contact management policy, the general policy of China and WHO is more or less the same. However, with the changes of epidemic situation and normalization management, China's management measures for close contacts have been gradually revised and improved. It is mainly reflected in defining the number and times of nucleic acid detection, so as to understand the outcome of close contacts as soon as possible. Through the close contacts tracking of suspected cases of COVID-19 in Chengdu, as of May 1, 2020, 986 close contacts in cities around Chengdu were brought into effective medical observation, and 7 were converted to confirmed cases (attack rate 0.71%), significantly lower than other cities in China (3.7%) (17) and Ireland (7.0%) (18). These results suggested that tracking and management of close contacts could effectively reduce the delay between infection and isolation, thus preventing the further spread of the virus.

During the COVID-19 pandemic, the impact of community environment is enormous, which has proved that early and strict isolation tracking was an effective strategy to limit clusters (19, 20). Close contact tracing mainly includes identification, listing, and tracking, and is an important aspect of epidemic control and often needs the help of all sectors of society (21). Besides, it is also a tedious task that requires a lot of human resources and cannot be fully implement in areas with widespread transmission (22–24). How to accurately identify and track management is still a difficult problem. At present, the most commonly used tracking technologies in the world are software and applications such as the CoV-SCR web-app (25, 26), which provides convenience for secret connection management. But there are still drawbacks. At the outbreak of the epidemic in Chengdu, the Chengdu Center for Disease Control and Prevention urgently developed a “Close Contact Information Management System” to dynamically identify cases and their close contacts. To some extent, this restrained the spread of the epidemic and the occurrence of second-generation cases.

Evidence so far showed that the transmission of COVID-19 occurred in the prodromal stage of mild illness of the infected person, and the interpersonal activities contributed to the spread of infection (8, 27). To curb the spread of the disease, the Chinese government has blocked the source city since January 23, 2020. However, the large-scale population movement during the Spring Festival may have contributed to the spread of the disease (7, 9). According to the big data of the Sichuan Mobile Network, from January 10 to January 20, as many as 22,000 people entered Chengdu from Wuhan. At the same time, COVID-19 carriers among them may have spread the virus to their contacts through work, travel, and gatherings (28), which undoubtedly increased the difficulty of epidemic prevention and control. The analysis of 986 close contacts found that the main contacts were passengers and relatives (80.42%), while the main modes were transportation and gatherings (71.70%), indicating that the key population to focus on for epidemic prevention and control should be co-passengers and relatives.

Similar to SARS and MERS, hospital transmission was a serious problem of COVID-19, or even worse. A recent retrospective study showed that 1,716 health workers were infected, accounting for 3.84% of the total cases (11). In this survey, medical personnel accounted for 2.03% of close contacts, and 9.13% of people became new close contacts through diagnosis and treatment and contact in the hospital. Nosocomial infections have greatly increased the burden on the health system and hindered early infections from obtaining timely medical support (29). In turn, it also suggested that the prevention and control of nosocomial infection may hinder the spread of the epidemic to a certain extent.

Our observational study has several limitations of importance for its interpretation, which mainly manifested in determining the possibility of recall bias and selection bias. First, tracking contacts through interviews are prone to recall bias, because individuals may not be able to recall events that occurred 14 days ago accurately, resulting in omissions or prolonging the finding time of some close contacts. Thus, close contact tracing systems or software seem to be particularly important (24). Besides, due to the existence of exclusion factors, it is indeed possible to determine the existence of selection bias, which may also have a certain impact on the attack rate. Fortunately, through the control of close contacts, the spread of the epidemic caused by close contacts has not been confirmed, minimizing the possibility of second-generation cases. At the same time, no association was found between the missing close contacts and previous cases. In addition, the data analyzed in this study seem to be out of date at this stage. To avoid this defect, we will analyze the latest data in our future research and compare the data differences between the two stages.

## Conclusion

Collectively, these findings illustrated that transportation and gatherings were the main ways to cause close contact infection. While focusing on co-passengers and relatives, we should also pay attention to nosocomial infection. Isolating close contacts at home or intensively for 14 days and monitoring their health every day could be part of the active case detection. We believe that if the public is encouraged to maintain their own contact list every day, this will help greatly to reduce the time and effort for contact tracing.

## Data Availability Statement

The raw data supporting the conclusions of this article will be made available by the authors, without undue reservation.

## Author Contributions

KY: conceptualization, methodology, formal analysis, and writing—original draft. JD: data curation and writing—original draft. LW: data curation and software. SJ: methodology and supervision. RL: software and validation. XT: conceptualization and project administration. ZL: data analysis and manuscript modification. All authors reviewed and agreed upon the final version of the manuscript.

## Funding

This study was supported by the Non-profit Central Research Institute Fund of Chinese Academy of Medical Sciences (2020-PT330-005) and Chengdu Municipal Science and Technology Bureau Key R&D Support Program Technology Innovation R&D Project (2020-YF05-00133-SN).

## Conflict of Interest

The authors declare that the research was conducted in the absence of any commercial or financial relationships that could be construed as a potential conflict of interest.

## Publisher's Note

All claims expressed in this article are solely those of the authors and do not necessarily represent those of their affiliated organizations, or those of the publisher, the editors and the reviewers. Any product that may be evaluated in this article, or claim that may be made by its manufacturer, is not guaranteed or endorsed by the publisher.

## Abbreviations

COVID-19: coronavirus disease 2019
WHO: World Health Organization
PHEIC: Public Health Emergency of International Concern.
